# Numerical modeling of heat transfer and pasteurizing value during thermal processing of intact egg

**DOI:** 10.1002/fsn3.257

**Published:** 2015-07-20

**Authors:** Behzad Abbasnezhad, Nasser Hamdami, Jean‐Yves Monteau, Hamed Vatankhah

**Affiliations:** ^1^ Department of Food Science and TechnologyFaculty of AgricultureIsfahan University of TechnologyIsfahan84156‐83111Iran; ^2^ UMR GEPEA (UA CNRS 6144 – SPI)ENITIAARue de la G_eraudi_ere, BP 82225NantesF‐44322France

**Keywords:** Egg, heat transfer, numerical modeling, pasteurization, *Salmonella enteritidis*, thermophysical properties

## Abstract

Thermal Pasteurization of Eggs, as a widely used nutritive food, has been simulated. A three‐dimensional numerical model, computational fluid dynamics codes of heat transfer equations using heat natural convection, and conduction mechanisms, based on finite element method, was developed to study the effect of air cell size and eggshell thickness. The model, confirmed by comparing experimental and numerical results, was able to predict the temperature profiles, the slowest heating zone, and the required heating time during pasteurization of intact eggs. The results showed that the air cell acted as a heat insulator. Increasing the air cell volume resulted in decreasing of the heat transfer rate, and the increasing the required time of pasteurization (up to 14%). The findings show that the effect on thermal pasteurization of the eggshell thickness was not considerable in comparison to the air cell volume.

## Introduction

Egg is a widely used as a highly nutritive food. Although it is available as different types such as frozen, powdered, and liquid, the consumer is tends to buy the intact egg (Denys et al. 2005; Ramachandran et al. 2011). The food products containing egg have a high risk of microbial spoilage due to being contaminated by different microorganisms a specially *Salmonella enteritidis* and *Escherichia coli* (Hema 2011; Hou et al. 1996). *Salmonella enteritidis* is the most heat resistant spoilage factor in egg (*D*_60_ *= *0.17 min*, z*_*value*_ *= *4.08°C). American food and drug association has recommended a 5D thermal processing for eggs based on *Salmonella enteritidis* (Abbasnezhad et al. 2014).

Industrial processes of pasteurization have to ensure the prolongation of food shelf life, while the quality of product should be preserved. The accomplishment of both purposes depends on process conditions with the assurance of the adequate temperature course during processing, where consideration of the temperature profiles within the product has great importance (Abbasnezhad et al. 2015). During the thermal process, the temperature inside the food depends on time as well as on the position inside the food system. The design of the thermal process is, therefore, always based on the temperature course in that position in food which receives the least intense heat treatment, known as the slowest heating point (SHP) or the slowest heating zone (SHZ). Heat transfer mechanisms in food are conduction for solid foods, natural convection, especially for low‐viscosity liquid foods, natural convection plus conduction for liquid foods with solid particles and natural convection followed by conduction for liquid foods containing starch or high viscosity modifiers (Chen and Ramaswamy 2002). Temperature profiles are determined using analytical and numerical solutions of partial differential equations governing the process (Kızıltaş et al. 2010). For realistic and more complicated heat transfer problems usually no analytic solution is available, and a numerical solution becomes mandatory (Ruiz‐Cabrera et al. 2014).

Thermal pasteurization of intact eggs has been studied vastly to decrease microbial population and to increase the shelf life, in recent years (Hou et al. 1996; Coimbra et al. 2006; Denys et al. 2004; Ferreira et al. 1997; Koen et al. 2007). Computational fluid dynamics (CFD) has been used to study heat transfer in various food processes (Koen et al. 2007; Barbosa‐Cánovas et al. 2011; Erdogdu et al. 2007; Ghani et al. 2001; Juliano et al. 2011; Mahesh and Kannan 2006; Norton and Sun 2006). Denys studied heat transfer and velocity profiles of egg shells filled with viscous liquids. The slowest heating point (SHP) was reported to be about the geometrical center of the samples (Denys et al. 2004). Denys developed a model based on CFD and experimental estimation tools to calculate surface heat transfer coefficient of eggs (Denys et al. 2003). Ramachandaran developed a three‐dimensional model to simulate the thermal pasteurization of eggs in stationary and rotational conditions of heat treatment. The results showed that rotation of eggs highly decreased the time needed for thermal treatment (Ramachandran et al. 2011). Fabbri studied thermal pasteurization of eggs in hot air currents, using CFD modeling. They also included the air cell of eggs in the model. The results showed that air cells have heat resistant characteristics (Fabbri et al. 2012). However, to our knowledge, no previous researchers have developed a general model for eggs pasteurization considering all parts of an egg such as white, yolk, and air cell.

The aims of the current work are: (1) to develop a three‐dimensional heat transfer model for intact egg pasteurization to allow prediction of temperature profile, SHZ location and pasteurization efficiency, and (2) to validate the theoretical model against experimental data.

## Model Development

### Geometry design and mesh generation

**Figure 1:**
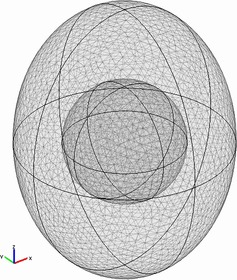
Designed and meshed geometry.

The geometry of the egg was designed as a 3D oval. The length and the radius of the oval were 6 and 4.5 cm, respectively. A sphere with radius of 1.6 cm was designed in the geometric center as the yolk. The air cell was designed according to the measured sizes of the real ones above and at the bottom of the shell as shown in Figure 1. The meshing was done in the “Uniform” trigonal type. The total count of trigonal elements was 288186.

### Assumptions of the model

The egg is placed into a water bath and its surface directly exposed to hot water, and heated by natural convection. The heating of the surface creates a temperature gradient, which is the origin of the driving force behind the heat transfer in the egg toward the center. Heat transfer inside the shell can be treated as heat conduction.

In order to simplify the problem, the following assumptions were considered:

1. Egg white and yolk are homogeneous and isotropic during pasteurization.
2. The initial temperature is constant and same in all eggs.
3. Thermophysical properties were used as functions of temperature, excluding the volumetric thermal expansion coefficient and specific heat.
4. Viscous dissipation was neglected because of low shear rates.
5. No‐slip boundary condition was assumed for velocity components relative to boundaries.
6. The natural convectional flow of fluid was assumed in the gravitational direction.
7. The moisture transfer was neglected during the process.

### Governing equations

Partial differential equations, related to governing natural convection motion of fluids (Navier stokes equations), and heat transfer phenomenon (energy equation) in a three‐dimensional *x*,* y*, and *z* coordinate system, are presented as (Ramachandran et al. 2011; Denys et al. 2003):

Continuity equation:

(1) $$\frac{\partial u_{x}}{\partial x}+\frac{\partial u_{y}}{\partial y}+\frac{\partial u_{z}}{\partial z}=0$$

Momentum equation:

(2) $$\left(\frac{\partial \rho V}{\sigma t}+▿.\rho V⊗V\right)=▿.\left(-p\delta +\mu \left(▿V+{\left(▿V\right)}^{t}\right)\right)+S_{M}$$

Energy equation:

(3) $$\frac{\partial T}{\partial t}+u_{x}\frac{\partial T}{\partial x}+u_{y}\frac{\partial T}{\partial y}+u_{z}\frac{\partial T}{\partial z}=\propto \left(\frac{\partial^{2}T}{\partial x^{2}}+\frac{\partial^{2}T}{\partial y^{2}}+\frac{\partial^{2}T}{\partial z^{2}}\right)$$

where *u* (m/s) is the velocity, *p* (Pa) is pressure, *ρ* (kg/m^3^) is the density, *μ* (m^2^/s) is the kinematic viscosity of the fluid, and *g* (m/s^2^) is the gravitational acceleration acting in the negative *z*‐direction, and *α* (m^2^/s) is the thermal diffusivity. To consider buoyancy, the force driving the convective motion of the egg white is the gravitational force comprised in the equations, and the variation of the density with temperature is expressed using the Boussinesq approximation that was very accurate to model the natural convection during thermal processing of egg bodies.

(4) $$S_{M}=\rho g$$

(5) $$\rho =\rho_{ref}\left[1-\beta \left(T-T_{ref}\right)\right]$$

where *T*_*ref*_ (°C) and *ρ*_ref_ (kg/m^3^) are the reference temperature and corresponding density, respectively, and *β* (1/K) is the volumetric thermal expansion coefficient of the liquid.

The dimensionless Rayleigh number, which measures the strength of buoyancy driven flows, was calculated. The Rayleigh number was lower than 10^8^ and showed laminar flow behavior during the process. (Erdogdu et al. 2007).

### Initial and boundary conditions

Yolk and white interface,

(6) $$T=T_{s},\;u=0,\;v=0\;for\;0\leq r\leq R.$$

Outside of eggshell,

(7) $$\frac{\partial T}{\partial r}=0,\;\frac{\partial u}{\partial r}=0\;v=0\;for\;0\leq z\leq H.$$

Inside of eggshell,

(8) $$\frac{\partial T}{\partial r}=0,\;u=0\;v=0\;for\;0\leq r\leq R.$$

*T*_*s*_ is the water bath temperature that was 333 for heating part.

Initially the egg yolk and white were at rest and at a uniform temperature

(9) $$T=T_{i},\;u=0,\;v=0\;at\;0\leq r\leq R,\;0\leq z\leq H.$$

The yolk wall was given as a coupled wall to the egg white for uniform heating.

### Pasteurizing value (*F*) calculation

The purpose of this calculations is to arrive at an appropriate process time under a given set of heating conditions to result in a given process lethality, or alternately to estimate the process lethality of a given process. In order to characterize the effect of temperature evolution on micro‐organism destruction at a given location during pasteurization, the so‐called pasteurizing value (*F*) can be calculated:

(10) $$F=\int_{0}^{t}10^{(T-T_{ref})/Z}dt$$

where *T*_*ref*_ is 60°C and *Z* is 4.08°C, based on the thermal resistance of *Salmonella enteritidis*. A 5D inactivation of the mentioned microorganism (*D*_*value *_= 0.17) is assumed to be a good thermal process (Hema 2011; American Egg Board, 2012).

### Model parameters

The inputs of the mathematical model are as follows: the water bath temperature was constant, product dimensions, density, component mass fractions, surface heat transfer coefficient of egg, initial product temperature, number of nodes in the space, heating and cooling, and time of pasteurization. Based on these inputs, the model will determine values of temperature at each node for each time step. The thermophysical properties used in the model are shown in Table 1.

**Table 1:** Thermal properties of egg white, yolk, and egg shell, used in the modeling

		Value	*r* ^2^	Source
Thermal conductivity (W/m °C)	Yolk	*k* = 0.0008 *T* a + 0.395	0.998	(Abbasnezhad et al. 2014)
	White	*k* = 0.0013 *T* + 0.5125	0.991	(Abbasnezhad et al. 2014)
	Egg shell	2.25	–	(Denys et al. 2004)
Density (kg/m^3^)	Yolk	*ρ *= −0.0023 *T* ^2^ − 0.1386 *T* + 1037.1	0.997	(Abbasnezhad et al. 2014)
	White	*ρ *= −0.0041 T^2^ − 0.0115 *T* + 1043.3	0.995	(Abbasnezhad et al. 2014)
	Egg shell	2300	–	(Denys et al. 2005)
Specific heat capacity (J/kg °C)	Yolk	3120	–	(Denys et al. 2005)
	White	3800	–	(Denys et al. 2004)
	Egg shell	888	–	(Denys et al. 2004)
Surface heat transfer coefficient (h) (W/m^2^ °C)	–	2072^b^	–	(Abbasnezhad et al. 2014)
Viscosity (Pa s)	White	*η *= −0.0006 *T* + 0.0438	0.989	(Abbasnezhad et al. 2015)
	Yolk	*η *= −0.0118 *T* + 0.7915	0.996	(Abbasnezhad et al. 2015)

a *T* represents temperature in (°C).

b Calculated from data measured at 60°C.

### Numerical solution of the model

In this study, the resulting system of the above partial differential equations were solved by the “finite element” method by COMSOL Multiphysics 3.5a (COMSOL Inc., Burlington, MA). The solution of the equations was obtained in the “Direct UMFPACK” mode and time intervals were set with the help of the algorithm present in the software. The total process time was 2000 s and time step was 1 s. Maximum relative error of 10^−3^ was selected as the criterion for convergence relative to the tolerance window.

## Experimental Methodology

The experiments were carried out on intact fresh eggs. Eggs, not older than 3 days, bought from local stores and kept in 4°C until the time of experiments. The eggs were classified into three groups as big (heavier than 60 g), medium (55–60 g), and small (<55 g) (Atılgan and Unluturk 2008). The volume percent of air cells studied in this paper ranged from zero to 7%. The volume percent of the yolk, white, and shell were split according to Table 2.

**Table 2:** Average volume percent of egg components

	Yolk	White	Shell	Air cell
Volume percent	26.5	63	7.5	0–7

The eggs were placed at 20°C for 2 h before pasteurization. Pasteurization was carried out in a circulating water bath (Memmert, Germany) at 60°C for 35 min. The water bath used was equipped with an automatic temperature controller (±0.1°C). K‐type thermocouples (Omega, Stamford, CT, USA) with 0.3 mm diameter (±0.1°C) were placed in egg and water bath, to measure the temperature inside the egg and surrounding water. Before processing, a calibrated thermocouple was inserted through a hole into the egg (thermocouple tip positioned from the top surface of the eggshell to 40 mm inside the egg in a central axial vertical position) and sealed with glue. Thermocouples were connected to a temperature data logger (Datalog 20, AOIP, Evry, France) with 15 channels to carry out the measurements. The egg was immersed in a water bath and the temperature data acquisition was started. The data were used to validate the finite‐element model. To determine the thermocouple probe position, after completion of the thermal process, the temperature of the water bath was raised and the eggs were cut and the exact location of the probe determined.

## Results and Discussion

### Model validation

**Figure 2:**
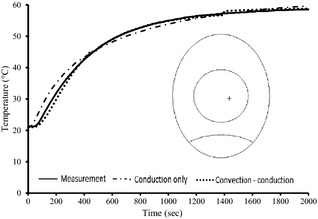
Comparison of egg temperature profiles between measurement and CFD predictions for convection‐ conduction and conduction only heating. Positions of the thermocouple probes and different mechanism for heat transfer are indicated. Exact location of thermocouple was (0.001, 0.006, 0.001).

The model validation was done by comparing the predicted temperature history with experimental results. Figure 2 shows a typical graph of the comparison between the experimental and predicted temperature evolutions for both the supposed heat transfer mechanisms (conductive or conductive–convective) in the egg white. Considering a natural convection‐conduction mode in the white and conduction mode in the yolk, a better agreement is obtained (*RMSE *= 0.0023 and *r* ^2 ^= 0.991) than a conductive mode for both (*RMSE *= 1.256 and *r* ^2 ^= 0.89). It can be concluded that the assumptions applied to develop the model (conductive–convective and conductive heat transfer in the white and yolk, respectively, and other assumptions) describe the heat transfer mechanisms well during egg pasteurization (Ramachandran et al. 2011; Denys et al. 2004).

### Effect of air cell on the slowest heating zone (SHZ) and flow patterns

**Figure 3:**
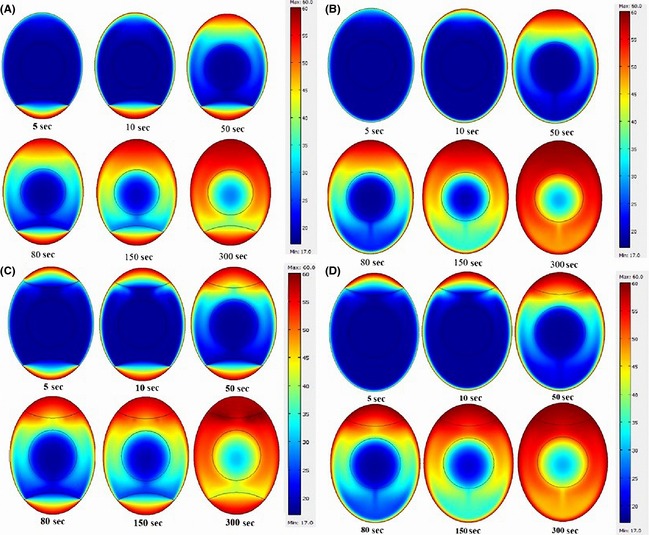
Temperature distribution of eggs having different air cell locations, during pasteurization process. (A) The air cells located at the bottom (B) Absence of air cell (C) two air cells, on top and bottom (D) The air cells located at the top.

After validation, the developed model was used to determine the SHZ of the egg during pasteurization. Figure 3 represents the results of the simulation for the intact eggs, without and with the air cell in three different positions, heated in a water bath at 60°C. As can be seen, the air cell is capable of influencing the heat transfer during thermal pasteurization of the intact eggs. The SHZ of the eggs with an air cell, be located in the bottom of the shell, was between the geometric center of the sample and upper zone of the air cell, as shown in Figure 3A. Assuming the absence of air cell, the SHZ would locate in the geometric center zone (Fig. 3B). The SHZ of the eggs with two air cells, on top and bottom, was in the geometric center (Fig. 3C). The air cells located at the top of the egg resulted in the displacement of the SHZ toward the center of the yolk (Fig. 3D).

**Figure 4:**
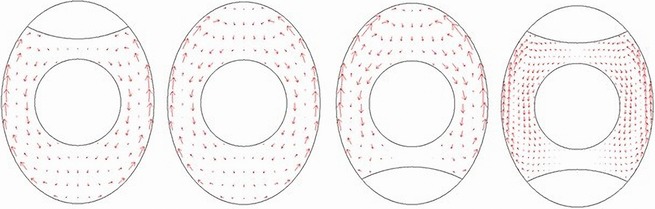
White flow pattern of eggs having different positions of air cells at 50 s of the thermal process in 60°C.

Figure 4 shows that the fluid in eggs is warmed up and flows upward in the boundary layer near the lateral eggshell. The warm liquid coming from the boundary layer almost stagnates at the top of egg. In fact, according to the mass balance terms, it flows slowly inward and downward. The area with downward flow is much greater than the area with the upward flow as can be seen on the horizontal cross section (Figure 4). As the liquid descends, its temperature decreases because of conduction and mixing with the lower and colder liquid layers. Arriving near the bottom, the liquid moves again toward the lateral wall and begins a new cycle. It can be seen that existence of air cells results in flow pattern change in the egg white. The egg with the air cell at the bottom has a stronger flow movement than with the air cell at the top and bottom of the egg.

**Figure 5:**
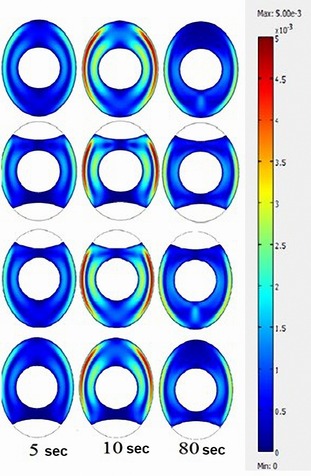
Velocity contours of models that having different air cell positions at 5, 10, and 80 s of the thermal process.

As shown in Figure 5, the natural convectional flow streams may decrease during the heat treatment, due to the fact that at the initial stages of the process, the high temperature gradient of the hot water and the inner fluid reinforce the buoyancy driven flows.

### Effect of the air cell volume and shell thickness on pasteurizing value

**Figure 6:**
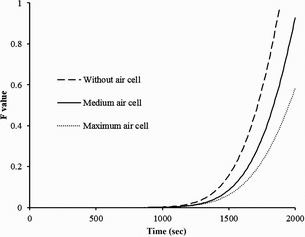
Effect of different air cell sizes on pasteurization efficiency.

Pasteurizing value can be calculated using the predicted temperatures at the SHP of the egg. Figure 6 shows the impact of air cell volume of the egg on the pasteurizing value. The results of simulation studies on the required heating time to achieve *F*_*value *_= 2.85 min, for three egg models with different air cell volumes (0, 3, and 7%) at 60°C, demonstrated that the eggs with larger air cells need more processing time to obtain the same *F*_*value*_. The required time for 0, 3, and 7% of the air cell volume in egg was 1680, 1770, and 1920 s, respectively. The SHP of the eggs with a medium air cell was between the geometric center of the sample and the upper zone of the air cell. SHP of the eggs with a minimum percent of air cell, the SHP located in the geometric center zone, and the SHP of the eggs with a maximum air cell volume were the upper zones of the air cell.

The heating times to obtain an equal pasteurization value (*F*_60_°C = 2.85 min) with different egg shell thicknesses were simulated. For thicknesses of 0.175, 0.4, and 0.25 mm, the required times were 1670, 1695, and 1710 s, respectively. This shows that an increase in shell thickness leads to a reduction in heat transfer and consequently an increase in the required heating time to achieve a satisfactory *F*‐value.

## Conclusions

A numerical model was developed to simulate 3D heat transfer in the intact egg to predict the local temperature and *F*_*value*_ during pasteurization. The model accommodates the effects of air cell volume and temperature‐dependent variables such as density and thermal conductivity. The model was validated by comparison of the experimental temperature profiles during pasteurization of the egg with the predicted values. The main results that can be drawn from this study are the following: (1) the position and size of air cells affect the heat transfer patterns and characteristics during egg pasteurization; (2) the thickness of the eggshell affects the required pasteurization time; (3) the effect of eggshell thickness on the required pasteurization time was less than the air cell volume; and (4) the model is useful to describe the heat transfer phenomenon during egg pasteurization.

## Conflict of Interest

The authors do not have any conflict of interest.
